# Association between long-term weight-change trajectory and cardiovascular disease risk by physical activity level

**DOI:** 10.1038/s41598-022-17765-0

**Published:** 2022-08-12

**Authors:** Hye Ah Lee, Hyesook Park

**Affiliations:** 1 Clinical Trial Center, Ewha Womans University Mokdong Hospital, 1071, Anyangcheon-ro, Yangcheon-ku, Seoul, 07985 Republic of Korea; 2 Department of Preventive Medicine, College of Medicine, Ewha Womans University, Seoul, Republic of Korea; 3 Graduate Program in System Health Science and Engineering, Ewha Womans University, Seoul, Republic of Korea

**Keywords:** Epidemiology, Risk factors, Cardiology

## Abstract

Using data from the Korean Genome and Epidemiology Study, we identified weight-change patterns during midlife using a group-based trajectory model, and evaluated their associations with the incidence of cardiovascular disease (CVD). At baseline, there were 8774 CVD-free participants. Group-based modeling was used to analyze patterns of weight change over about 16 years. Using multiple model, we evaluated the association between weight-change patterns and CVD risk. During the follow-up period, 741 new CVD cases were identified. The weight-change patterns were characterized as ‘gradual weight gain’, ‘stable weight’, ‘slight weight loss’, and ‘gradual weight loss’. The association between weight-change patterns and CVD risk differed depending on the level of physical activity (PA) at baseline (*p*_interaction_ < 0.05). Compared with the stable-weight group, the risk of all CVD (HR 2.5, 95% CI 1.5–4.3) and non-fatal CVD (HR 2.8, 95% CI 1.6–4.9) among the gradual-weight-loss group was apparent in the lowest PA quartile. In addition, on average, a decrease in skeletal-muscle-mass (SMM) levels was observed during the follow-up period, but the decrease in SMM in the gradual-weight-loss group was greater than in the gradual-weight-gain group. Our findings show that gradual weight loss was associated with CVD risk, which was dependent on PA levels.

## Introduction

Over the past two decades, cardiovascular disease (CVD) mortality has declined markedly worldwide^1^, but the burden of CVD on public health remains high, accounting for 330 million years of life lost and 35.6 million years lived with disability in 2017^2–4^. The J-shaped or U-shaped association between body mass index (BMI) and CVD risk is well established in the literature^5,6^; however, several studies have focused on the effect of weight variability on health risks^7–10^. These studies hypothesized that weight changes across adulthood are common and the impact on health varies^7,11^. A study using the US National Health and Nutrition Examination Survey (NHANES) 1988–2004 data observed a U-shaped association between weight change over the previous 10 years and heart disease mortality among participants 40 years of age or older^7^. In the National Health Insurance System-Korean National Health Screening Program study that included ten million participants aged 20 and older, both weight gain and weight loss of more than 5% over 4 years were associated with an increased risk of ischemic stroke^8^. In another aspect, it has been reported that weight cycling (i.e., weight fluctuation), which means multiple repetitions of weight loss and regain, can induce fluctuations in cardiometabolic risk factors and is also associated with CVD mortality and morbidity^12^. Despite this progress, studies evaluating the relationship between weight change and the incident CVD risk in the general population are still lacking.

Regarding weight-change assessment, a group-based trajectory model was introduced to identify various trajectory patterns in longitudinal data^13^ and used in epidemiological studies^14,15^. However, previous studies have used data collected through two weight measurements^8,11,16^ or recall^7,10,11^. To evaluate the variability in repeatedly measured weight, some studies have calculated the coefficient of variation and the root mean square error^12^. It is possible to evaluate the degree of fluctuation, but it is difficult to identify the pattern, and there is no standardized definition for measurement of weight fluctuation. Thus, studies evaluating the trajectory patterns of weight change throughout the lifespan are limited. In addition, there remain questions regarding the obesity paradox in health risk, as studies have shown an increased health risk associated with weight loss compared to weight maintenance, as well as some beneficial health effects of being overweight^17,18^. It has been suggested that muscle reduction may contribute to the increased risk among the weight-loss group^9^; however, muscle-mass variability was not assessed along with weight change. It has also been suggested that sarcopenic obesity may have a higher CVD risk than sarcopenia and obesity alone^19^. In this regard, physical activity (PA) may be closely related to weight control^20^ or CVD risk^21^. One study noted that the effect of the trajectory of BMI on atrial fibrillation could vary depending on PA^22^. Therefore, we tried to evaluate how PA affects the relationship between weight trajectory and CVD risk.

Therefore, this population-based study identified weight-change patterns during midlife using a group-based trajectory model and evaluated the association between weight-change patterns and incident CVD controlling for several covariates. Additionally, changes in the skeletal-muscle mass (SMM) associated the identified trajectory patterns were assessed to account for potential causal pathways.

## Methods

### Data and study subjects

This study was conducted using data from the community-based cohort (Ansung-Ansan cohort) in the Korean Genome and Epidemiology Study (KoGES). Detailed information regarding the KoGES has been published elsewhere^23^. This community-based cohort study began in 2001–2002 to investigate risk factors for chronic diseases among Koreans. This prospective cohort included residents (aged 40–69 years) living in two communities in Gyeonggi Province: Ansung, a rural region (n = 5018), and Ansan, an industrial region (n = 5012). The 10,030 participants completed the baseline survey in 2001–2002. Biennial follow-up assessments were conducted by trained technicians and interviewers and are still ongoing. The follow-up assessments include questionnaires, anthropometric measurements, blood sampling (collected after overnight fasting), urine tests, and biomarker measurements. This study included data up to the eighth follow-up (conducted in 2017–2018; follow-up rate = 61.4%). There were no significant differences between follow-up participants and non-participants, except in smoking status, total calories, and fasting blood glucose levels^23^.

In this study, participants with a history of cancer, myocardial infarction, coronary artery disease, cerebrovascular disease, or congestive heart failure at baseline were excluded (n = 410). In addition, participants had to have completed at least one follow-up survey to be included in the analysis. After exclusions, this study included 8774 participants (4161 males and 4613 females). The excluded individuals were slightly older, had a higher prevalence of diabetes and hypertension, and had lower physical activity levels than those who were included; however, there were no differences in mean BMI, weight, or current-smoking percentage (data not shown). Our study followed the STROBE guidelines. The study was conducted according to the guidelines of the Declaration of Helsinki and the study protocol was also approved by the Institutional Review Board (IRB) of Ewha Womans University Hospital (IRB no. EUMC 2017-06-041/EUMC 2021-03-008). Review board requirement for written informed consent was waived by the IRB of Ewha Womans University Hospital because this study used an anonymous dataset.

### Cardiovascular diseases assessment

As an outcome, newly identified cases during the follow-up period were considered. For the conditions diagnosed by physicians, self-reported questionnaires for myocardial infarction, coronary artery disease, cerebrovascular disease, and congestive heart failure were used to determine the incidence of non-fatal CVD. If a medical diagnosis was confirmed during follow-up, the event was considered to have occurred on the date of follow-up; the follow-up time was then calculated. Thus, follow-up commenced on study entry and ended when the physician-diagnosed disease was confirmed or on the date of the last follow-up. Fatal CVD was determined based on the cause of death of each subject as of December 31, 2019 by linking the death certificate data from the Korean National Statistical Office and cohort data. Fatal CVD was defined as codes of I00-I79 based on the International Classification of Disease, 10th Revision^24^, and confirmed deaths due to myocardial infarction (I21–I22), coronary artery disease (I20–I25), cerebrovascular disease, (I60–69) and congestive heart failure (I50). The validity of the data was assessed by comparing self-reported diagnoses with the diagnoses reported in the medical records, and 93% agreement between self-reported diagnoses and the medical records was observed among 30 cases^25^.

### Weight-change and body-composition assessment

The average number of measurements of weight for participants was 6.7 ± 2.6. Using weight values before CVD development, bodyweight change was calculated by subtracting baseline weight from the weight at each follow-up time point.

Total skeletal-muscle mass (SMM) and body fat mass were measured using multi-frequency bioelectrical impedance analysis (MF-BIA; InBody 3.0; Biospace, Seoul, Korea). Body composition was measured at each follow-up and data were collected.

### Covariates

Several demographic factors collected at baseline were included as covariates including age, sex, study region (industrial/rural), and educational level at baseline. Additional potential risk factors for CVD including body mass index (BMI) (continuous), current smoking, alcohol intake (no alcohol, < 15 g/day, 15–24.9 g/day, ≥ 25 g/day)^27^, and sex-specific quartile of physical activity (PA) at baseline were also considered. The intensity and duration of PA over the past year were assessed using the metabolic equivalent of task (MET)-hours per week based on the International Physical Activity Questionnaire^28^. Additionally, since several prevalent conditions are associated with weight change and CVD risk, diabetes, hypertension, dyslipidemia, and arthritis were considered covariates. The condition was defined taking into account clinically relevant data or self-reported information for physician-diagnosed illnesses, collected as part of the baseline survey. Using clinically related data, diabetes was defined as a fasting blood glucose level ≥ 126 mg/dL, and hypertension was defined as a systolic or diastolic blood pressure of 140/90 mmHg or higher. Dyslipidemia was defined as triglyceride levels > 150 mg/dL or high-density lipoprotein-cholesterol levels < 40 mg/dL for men and < 50 mg/dL for women.

### Statistical analysis

All statistical analyses were conducted using SAS software (ver. 9.4; SAS Institute, Cary, NC, USA). Two-tailed p-values < 0.05 were considered statistically significant.

**Figure 1:**
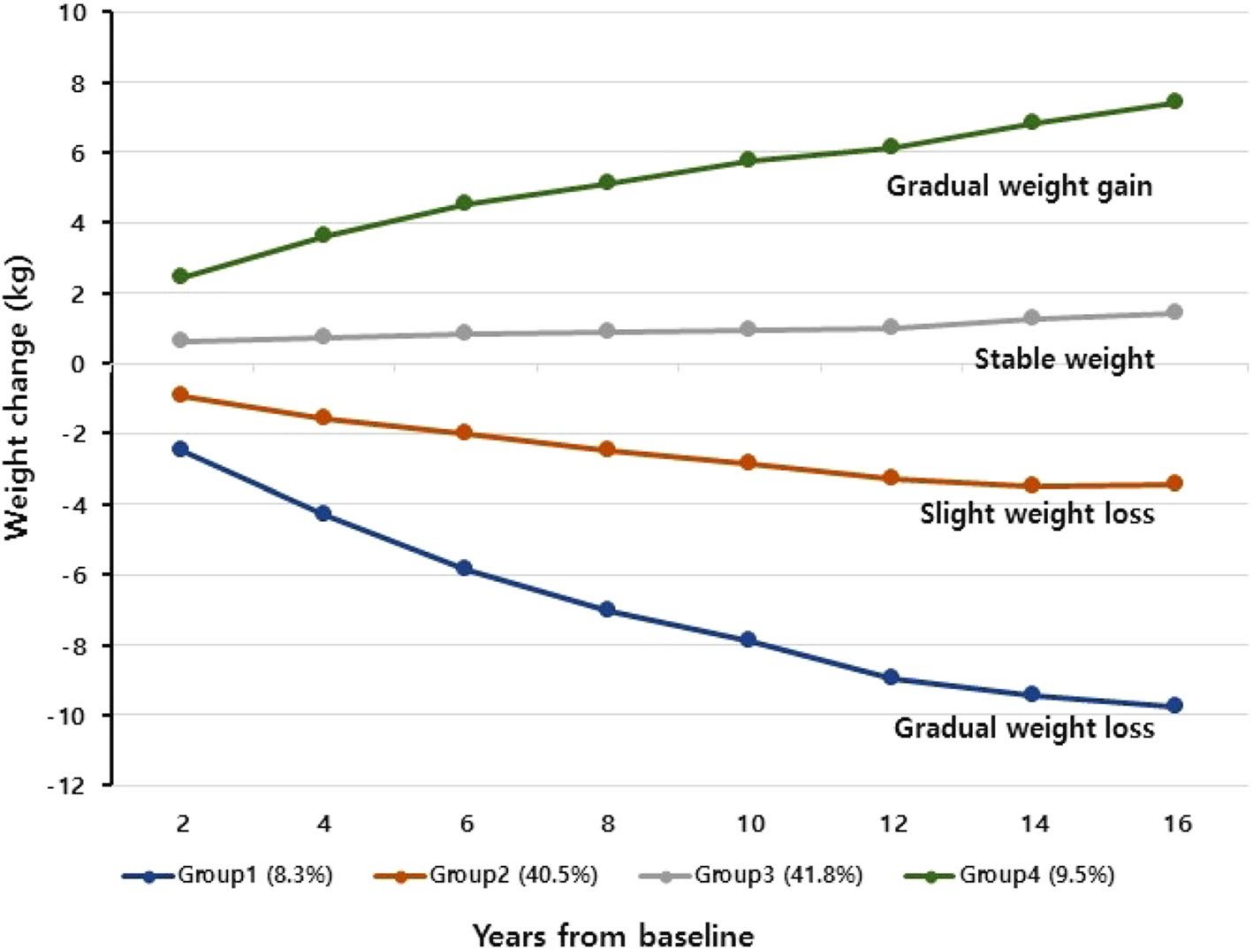
Weight changes by trajectory group derived from group-based modeling over the 16-year study duration. The solid line indicates the estimated average value.

Participants were categorized into distinct subgroups based on their specific pattern of weight change over time using the Proc Traj procedure in SAS software for group-based trajectory modeling^26^. A group-based trajectory model is a statistical technique for longitudinal data^13^ that has several advantages, including the discovery of unexpected and potentially meaningful trajectories and graphical representation of the results for better understanding^13^. Each trajectory can be specified individually, allowing the best-fitting polynomial form. The optimal fit model is chosen based on Bayesian Information Criterion (BIC) values, with each trajectory containing at least 5% of the predicted sample size^12^. To compare two models with different numbers of groups, we calculated the logged Bayes factor (2 × ΔBIC). The five-pattern model did not satisfy the group size requirements. Therefore, the four-pattern model was selected as the optimal model based on the logged Bayes factor (eTable 1). We then refined the four-pattern model until the highest polynomial coefficient for each trajectory group was statistically significant. Therefore, the highest polynomials for each group were quadratic, quadratic, linear, and quadratic, respectively (Fig. 1). For the selected model, based on the highest posterior probability, each individual was assigned to a specific trajectory group. The probability of belonging to a particular group can depend on the covariates, which can affect the model coefficients. Thus, sex and age were included in the model as covariates. The four groups were named ‘gradual weight loss’, ‘slight weight loss’, ‘stable weight’, and ‘gradual weight gain’ based on the features of their weight-change patterns.

Basic group characteristics were summarized as means with standard deviations for numerical data and number of participants with percentages for categorical data. Differences in basic characteristics among groups were tested using a generalized linear model and Chi-square test. Basic characteristics with significant differences between groups were included as covariates in the analysis.

The association between weight-trajectory patterns and CVD risk was evaluated using Cox proportional hazard regression modelling. We evaluated whether the influence of the trajectory patterns differed by PA quartile regarding CVD risk and observed significant interactions (*p*_interaction_ < 0.05). Thus, subsequent analyses were stratified by the PA quartile. The relationship between trajectory patterns and the CVD risk was estimated using hazard ratios (HRs) with 95% confidence intervals (95% CI). The multiple regression model included sex, age, study region, educational level, BMI, smoking status, alcohol intake, any history of diabetes, hypertension, dyslipidemia, or arthritis at baseline, and skeletal muscle mass change during follow-up. Furthermore, multiple models were stratified by obesity at baseline (BMI ≥ 25.0 kg/m^2^ or BMI < 25.0 kg/m^2^) and sex. Additionally, associations were evaluated in subjects with low-muscle mass and high-fat mass that were defined using the median of SMM and body fat mass.

A mixed-model analysis was performed to estimate the relationship between weight-change patterns and SMM or body fat mass. A random-intercept model was constructed to include group (i.e., weight-change patterns), measurement time point, the interaction between group and measurement time point as fixed effects, as well as the covariates. Sex, age, study region, education level, physical activity (MET), current smoking, alcohol intake, disease history of diabetes, hypertension, dyslipidemia, and arthritis at baseline, height (the residual from a regression model in which height is the independent variable and SMM is the dependent variable at each follow up)^29^, mutually adjusted for body fat mass (in the SMM model), and SMM (in the body fat mass model) at each follow up were included as covariates. Covariance structure was determined according to BIC values. The results were indicated as least-square means with 95% CIs.

## Results

In this study, 47.4% of the participants were male and their average age was 52.1 years at baseline. Of the study participants, 25.5% were current smokers and 42.6% were obese (BMI ≥ 25.0 kg/m^2^). The best-fit model of weight-trajectory patterns is shown in Fig. 1. Four subgroups of weight trajectory were identified, including gradual weight gain (9.5% of participants), stable weight (41.8%), slight weight loss (40.5%), and gradual weight loss (8.3%). Over the 16-year study period, the average change in weight was + 7.4 kg in the gradual-weight-gain group, + 1.4 kg in the stable-weight group, − 3.4 kg in the slight-weight-loss group, and − 9.8 kg in the gradual-weight-loss group.

Table 1 outlines the differences in basic characteristics among weight-trajectory groups. Participants in the gradual-weight-gain group were younger, more educated, and had a lower BMI, but were more likely to be current smokers and high alcohol consumers than those in the gradual-weight-loss group. The weight loss group had a higher prevalence of hypertension, diabetes, dyslipidemia obesity, and a high proportion of the lowest PA quartile. Average SMM and body fat mass also differed among groups.

**Table 1:** Basic characteristics of weight-trajectory groups.

	Weight-change groups								*P*
	Gradual weight loss (n = 725, 8.26%)		Slight weight loss (n = 3550, 40.46%)		Stable weight (n = 3665, 41.77%)		Gradual weight gain (n = 834, 9.51%)		
Age (years)	55.44	9.04	54.16	8.86	50.03	8.21	49.12	8.27	< 0.001
**Sex (%)**									
Male	310	42.76	1689	47.58	1682	45.89	480	57.55	< 0.001
Female	415	57.24	1861	52.42	1983	54.11	354	42.45	
**Rural region (%)**									
Yes	394	54.34	1901	53.55	1790	48.84	419	50.24	< 0.001
No	331	45.66	1649	46.45	1875	51.16	415	49.76	
**Education level (%)**									
Did not graduate high school	444	61.67	2148	61.04	1880	51.72	417	50.48	< 0.001
Graduated high school	190	26.39	948	26.94	1212	33.34	297	35.96	
Some college or higher	86	11.94	423	12.02	543	14.94	112	13.56	
**Sex-specific quartile of PA (%)**									
Q1	156	22.03	702	20.40	731	20.74	160	20.08	0.0594
Q2	200	28.25	908	26.38	969	27.49	202	25.35	
Q3	172	24.29	877	25.48	964	27.35	233	29.23	
Q4	180	25.42	955	27.75	861	24.43	202	25.35	
**Prevalent diseases (%)**									
DM	121	16.69	368	10.37	201	5.48	60	7.19	< 0.001
HTN	355	48.97	1372	38.65	1035	28.24	222	26.62	< 0.001
Dyslipidemia	502	69.24	2056	57.92	1952	53.26	348	41.73	< 0.001
Arthritis	38	5.24	154	4.34	144	3.93	28	3.36	0.2352
Current smoker (%)	149	20.67	822	23.47	915	25.31	326	39.66	< 0.001
**Alcohol intake (%)**									
No alcohol	426	60.25	1864	54.00	1872	52.81	376	47.12	< 0.001
< 15 g/day	156	22.07	952	27.58	996	28.10	232	29.07	
15–24.9 g/day	51	7.21	237	6.87	238	6.71	55	6.89	
≥ 25 g/day	74	10.47	399	11.56	439	12.38	135	16.92	
BMI (kg/m^2^)	26.72	3.11	24.83	3.10	24.11	2.98	23.65	3.02	< 0.001
**Obesity status (%)**									
BMI < 25.0 kg/m^2^	204	28.14	1907	53.72	2340	63.85	587	70.38	< 0.001
BMI ≥ 25.0 kg/m^2^	521	71.86	1643	46.28	1325	36.15	247	29.62	
Weight (kg)	68.20	10.49	63.29	10.23	61.87	9.73	62.39	9.70	< 0.001
Skeletal muscle mass (kg)	44.94	8.49	43.31	8.14	43.13	7.95	44.58	7.88	< 0.001
Body fat (kg)	20.46	5.66	17.36	5.52	16.21	5.20	15.19	5.53	< 0.001

*PA* physical activity, *DM* diabetes mellitus, *HTN* hypertension, *BMI* body mass index.

**Figure 2:**
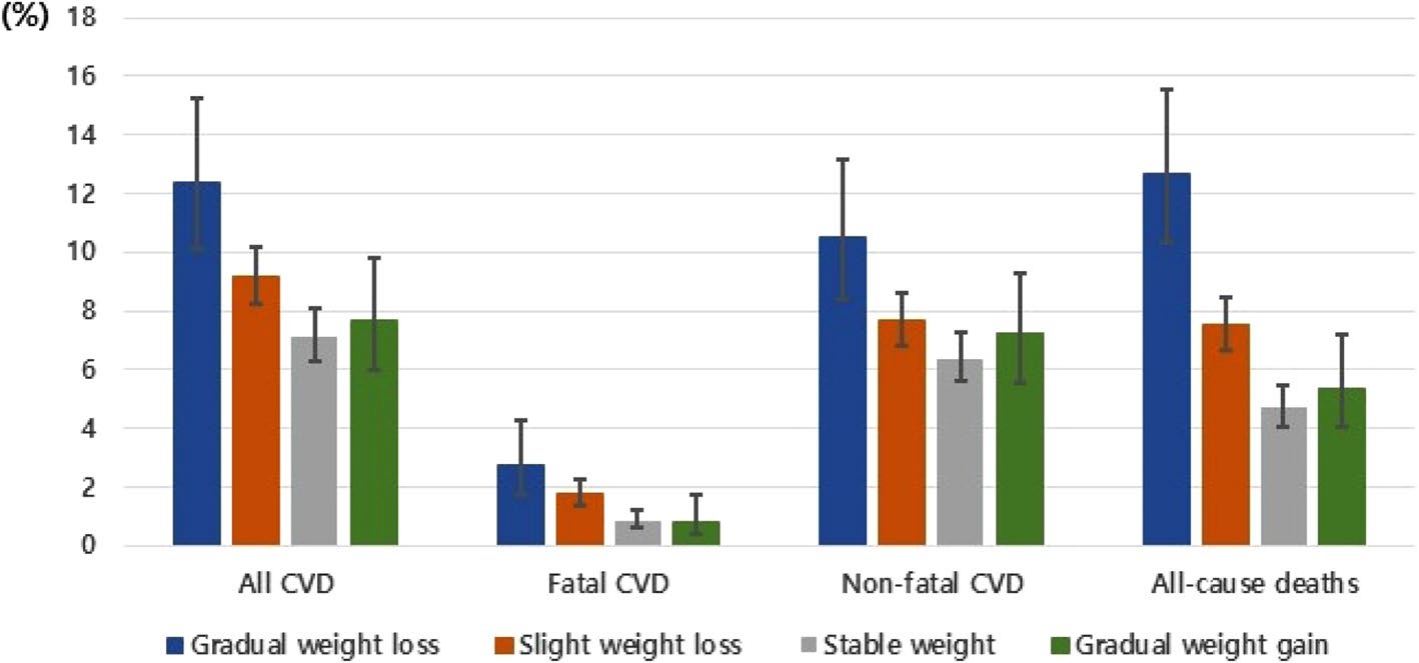
Incident cases proportion (%) with 95% CI among weight-trajectory groups. *CVD* cardiovascular disease, *95% CI* 95% confidence interval.

During the 16-year follow-up period, 741 new CVD cases (cumulative incidence = 8.4%) and 577 deaths due to all causes were identified. Of the cardiovascular-related diseases, cerebrovascular disease was most common (n = 351, cumulative incidence = 4.0%), followed by coronary artery disease (n = 278, cumulative incidence = 3.2%) and myocardial infarction (n = 122, cumulative incidence = 1.4%). The distribution of incident cases according to weight trajectory patterns is provided in Fig. 2. Overall, gradual weight loss group showed a higher incidence of CVD and all-cause mortality. Tables 2 and 3 show the results of the relationship between weight trajectory and incident CVD and all-cause mortality by physical activity level. Compared to the stable-weight group, the increased risk of CVD among the gradual-weight-loss group was apparent in the lowest PA quartile, and this significance was maintained in all CVD and non-fatal CVD even after adjusting for various covariates. In the third quartile of PA, the gradual-weight-loss group tended to have a lower risk of all CVD, but did not reach significance. In addition, gradual-weight-loss group was associated with a higher HR of all-cause mortality in the lowest quartiles of PA. To reduce bias attributable to missing data, we performed multiple imputations using five iterations of the fully conditional specification algorithm. We found that individuals with low PA levels who were in the gradual weight loss group still associated with a high risk of CVD (HR 2.21, 95% CI 1.30–3.75 for all CVD; HR 2.38, 95% CI 1.42–4.00 for non-fatal CVD) and a higher risk of all-cause death (HR 1.93, 95% CI 1.05–3.54) (data not shown).

**Table 2:** Association between weight trajectory and incident CVD and all-cause mortality by physical activity level (crude model).

Outcome	Weight-change groups	Sex-specific quartiles of PA											
		PA Q1 (low)			PA Q2			PA Q3			PA Q4 (high)		
		Event/N	HR	95% CI	Event/N	HR	95% CI	Event/N	HR	95% CI	Event/N	HR	95% CI
All CVD	Gradual weight loss	31/156	**2.74**	**1.74–4.30**	23/200	**1.94**	**1.19–3.16**	11/172	0.79	0.42–1.48	22/180	1.37	0.85–2.22
	Slight weight loss	62/702	1.30	0.89–1.89	75/908	**1.53**	**1.08–2.17**	64/877	0.97	0.69–1.36	111/955	**1.46**	**1.08–1.96**
	Stable weight	50/731	1.00		55/969	1.00		73/964	1.00		71/861	1.00	
	Gradual weight gain	8/160	0.64	0.30–1.35	17/202	1.31	0.76–2.26	19/233	1.05	0.63–1.74	18/202	1.09	0.65–1.84
Fatal CVD	Gradual weight loss	8/156	**4.93**	**1.85–13.13**	4/200	**6.63**	**1.48–29.64**	2/172	1.43	0.30–6.73	4/180	1.84	0.58–5.77
	Slight weight loss	12/702	1.60	0.65–3.90	8/908	2.86	0.76–10.80	12/877	1.66	0.68–4.06	26/955	**2.20**	**1.09–4.45**
	Stable weight	8/731	1.00		3/969	1.00		8/964	1.00		11/861	1.00	
	Gradual weight gain	2/160	1.15	0.24–5.42	0/202	NA	NA	1/233	0.51	0.06–4.10	4/202	1.56	0.50–4.89
Non-fatal CVD	Gradual weight loss	27/156	**2.89**	**1.78–4.71**	20/200	**1.83**	**1.09–3.06**	9/172	0.72	0.36–1.45	18/180	1.38	0.82–2.34
	Slight weight loss	51/702	1.29	0.86–1.94	67/908	**1.43**	**1.00–2.06**	54/877	0.95	0.66–1.37	90/955	**1.41**	**1.02–1.95**
	Stable weight	42/731	1.00		53/969	1.00		65/964	1.00		62/861	1.00	
	Gradual weight gain	7/160	0.67	0.30–1.49	17/202	1.35	0.78–2.34	19/233	1.14	0.69–1.91	15/202	1.03	0.58–1.80
All-cause death	Gradual weight loss	22/156	**3.49**	**2.02–6.02**	21/200	**2.90**	**1.70–4.98**	15/172	**2.39**	**1.31–4.37**	29/180	**2.31**	**1.49–3.59**
	Slight weight loss	52/702	**1.78**	**1.14–2.78**	52/908	**1.55**	**1.01–2.37**	53/877	**1.63**	**1.07–2.49**	99/955	**1.45**	**1.06–1.99**
	Stable weight	31/731	1.00		36/969	1.00		36/964	1.00		63/861	1.00	
	Gradual weight gain	8/160	1.19	0.55–2.59	6/202	0.80	0.34–1.90	8/233	0.91	0.42–1.96	23/202	1.57	0.98–2.54

Significant values are in bold.

*CVD* cardiovascular disease, *PA* physical activity, *HR* hazard ratio, *95% CI* 95% confidence interval.

**Table 3:** Adjusted hazard ratio of incident CVD and all-cause mortality associated with weight trajectory by physical activity level.

Outcome	Weight-change groups	Sex-specific quartiles of PA											
		PA Q1 (low)			PA Q2			PA Q3			PA Q4 (high)		
		N	HR	95% CI	N	HR	95% CI	N	HR	95% CI	N	HR	95% CI
All CVD	Gradual weight loss	143	**2.53**	**1.50–4.29**	183	1.28	0.73–2.24	166	0.55	0.28–1.09	151	1.40	0.83–2.38
	Slight weight loss	647	1.11	0.73–1.69	850	1.14	0.78–1.65	822	0.74	0.52–1.07	858	**1.44**	**1.05–1.96**
	Stable weight	671	1.00		912	1.00		909	1.00		779	1.00	
	Gradual weight gain	144	0.62	0.28–1.39	196	1.17	0.67–2.05	218	1.07	0.63–1.82	182	0.78	0.44–1.41
Fatal CVD	Gradual weight loss	143	1.99	0.58–6.84	183	1.76	0.25–12.60	166	0.33	0.04–2.89	151	1.10	0.29–4.14
	Slight weight loss	647	0.79	0.27–2.35	850	1.04	0.24–4.48	822	1.05	0.37–2.98	858	1.56	0.74–3.31
	Stable weight	671	1.00		912	1.00		909	1.00		779	1.00	
	Gradual weight gain	144	0.82	0.10–7.07	196	NA	NA	218	0.71	0.08–6.05	182	1.30	0.35–4.81
Non-fatal CVD	Gradual weight loss	143	**2.77**	**1.56–4.91**	183	1.23	0.69–2.18	166	0.56	0.27–1.14	151	1.44	0.81–2.55
	Slight weight loss	647	1.14	0.72–1.79	850	1.09	0.74–1.59	822	0.74	0.51–1.08	858	**1.41**	**1.01–1.98**
	Stable weight	671	1.00		912	1.00		909	1.00		779	1.00	
	Gradual weight gain	144	0.73	0.32–1.65	196	1.20	0.68–2.12	218	1.16	0.68–1.97	182	0.75	0.40–1.41
All-cause death	Gradual weight loss	143	**2.05**	**1.05–4.02**	183	1.24	0.61–2.49	166	1.43	0.71–2.90	151	1.63	0.99–2.66
	Slight weight loss	647	1.22	0.73–2.05	850	0.95	0.58–1.54	822	1.14	0.70–1.85	858	1.08	0.77–1.51
	Stable weight	671	1.00		912	1.00		909	1.00		779	1.00	
	Gradual weight gain	144	0.97	0.39–2.40	196	1.06	0.44–2.59	218	1.03	0.45–2.36	182	1.38	0.82–2.30

Significant values are in bold.

Hazard ratios with 95% confidence intervals were calculated with adjustment for sex, age, rural residence, educational level, body mass index, alcohol intake, current smoking, history of diabetes, history of hypertension, history of dyslipidemia, history of arthritis, and skeletal muscle mass change.

Significance are in bold.

*CVD* cardiovascular disease, *PA* physical activity, *HR* hazard ratio, *95% CI* 95% confidence interval.

In subgroup analysis, when stratified by obesity status at baseline, a high CVD risk in the lowest PA quartile among the gradual-weight-loss group remained in obese groups (eFigure 1). Although statistical significance was lost in the results stratified by sex, the gradual-weight-loss group in the lowest PA quartile showed a high CVD risk (eTables 2 and 3). In subjects with low muscle mass and high fat mass, a risk of all CVD in the gradual-weight-loss group was apparent in the lowest PA quartile, whereas a risk of all CVD among the gradual-weight-gain group was apparent in the highest PA quartile, compared with the stable-weight group (eTable 4).

**Figure 3:**
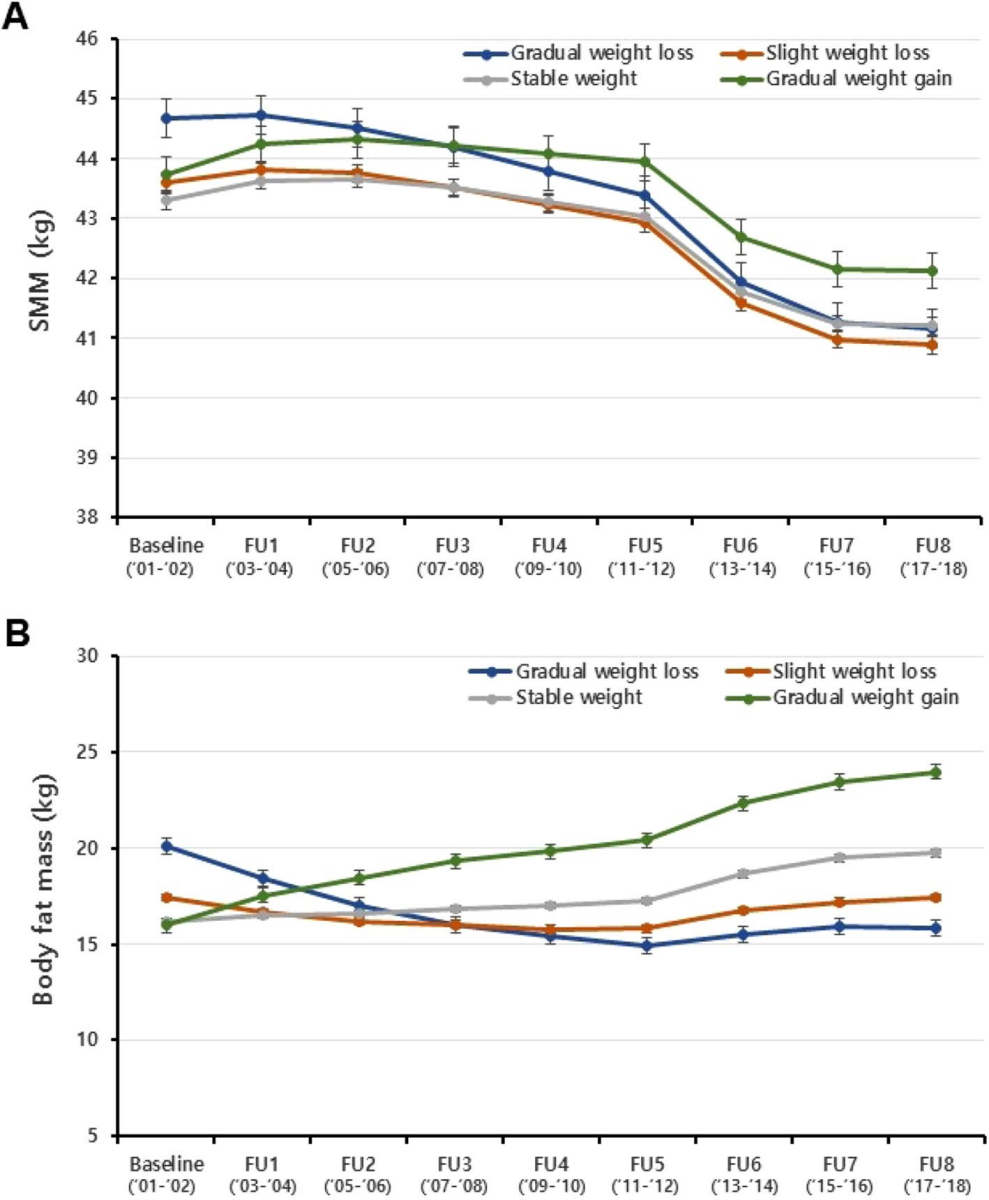
Changes in skeletal muscle mass and body fat mass among weight-trajectory groups. *SMM* Skeletal muscle mass, *FU* follow-up. (**A**) Changes in skeletal muscle mass among weight-trajectory groups, (**B**) Changes in body fat mass among weight-trajectory groups. Values are least-squared means with 95% confidence intervals. The least-squared mean change in SMM (**A**) or body fat mass (**B**) levels was estimated for each weight-trajectory group at each follow-up time point using a mixed model assuming a random intercept with an unstructured structure. Estimates were obtained from a model that included the trajectory group, follow-up time point, sex, age, rural residence, educational level, physical activity (MET), current smoking, alcohol intake, body mass index, prevalent diabetes, prevalent hypertension, prevalent dyslipidemia, prevalent arthritis at baseline, height (the residual from a regression model in which height is the independent variable and SMM is the dependent variable at each follow up), mutually adjusted for body fat mass (in the SMM model) and SMM (in the body fat mass model) at each follow up, and the interaction between trajectory group and follow-up time point.

The SMM or body fat mass variation among weight-trajectory groups is shown in Fig. 3. At baseline, the average SMM levels in the gradual-weight-loss group was higher than other groups, but mean SMM levels in the gradual-weight-loss group decreased throughout the follow-up period. The average body fat mass at baseline also showed statistically significant differences between weight-trajectory groups. Compared to the baseline values, SMM in the gradual-weight-loss group decreased by 3.5 kg (95% CI 3.4–3.7, *Bonferroni adjusted p* < 0.001), while SMM in the gradual-weight-gain group decreased by 1.6 kg (95% CI 1.5–1.7, *Bonferroni adjusted p* < 0.001). In terms of body fat mass, the gradual-weight-loss group showed an average decrease of 4.3 kg (95% CI 3.8–4.8, *Bonferroni adjusted p* < 0.001) from the baseline value, whereas the gradual-weight-gain group showed an average increase of 8.0 kg (95% CI 7.6–8.4, *Bonferroni adjusted p* < 0.001).

## Discussion

Using longitudinal data measured at multiple time points, four distinct trajectory patterns of weight change over a 16-year period were identified by group-based modeling, and their association with the incident CVD and all-cause mortality was evaluated. Individuals with gradual weight loss but low PA levels had a higher risk of incident CVD, especially in non-fatal CVD. In addition, gradual weight loss was also associated with a higher risk of death in the lowest quartiles of PA. With aging, a decrease in SMM was observed in all subjects, but a decrease in SMM was evident in the gradual weight loss group.

Recent studies assessing the relationship between weight change and health risks have shown that weight gain or loss is associated with heart-disease mortality^7^, incident atrial fibrillation^30^, acute myocardial infarction^16^, and ischemic stroke^10^. However, these studies have reported mixed results. Differences among study participants, study durations, and weight-change assessment methods may have contributed to these inconsistent findings. Furthermore, several studies have reported that age and the duration of weight change affect the health risk^7,10^. In the Atherosclerosis Risk in Communities (ARIC) study, long-term (30 years) weight loss from young adulthood had no effect on the incidence of CVD, whereas short-term weight loss over 3 years was associated with an increased risk for CVD in middle-aged individuals^10^. On the other hand, early weight gain over a long interval was significantly associated with increased risk of CVD compared to weight gain over a short interval^10^. Similar trends have been reported in a mortality study^7^. These findings imply that the health risk associated with weight change should be interpreted in the context of age and the duration of the weight change. In the present study, weight changes in middle-aged adults (40–69 years at baseline) were observed over 16 years, and weight loss was associated with CVD risk depending on the level of PA.

Studies evaluating the effects of weight gain on health risk have shown mixed results. A Korean population study on weight change and the incident risk of ischemic stroke showed that weight gain of more than 5% increased the risk of ischemic stroke by 6% (95% CI 1.05–1.08)^8^, while other studies have shown the reverse association^9^, and no association^22^. The pathological pathways associated with weight gain may be attributed to poor metabolism (i.e., inflammation^14^ and cardiometabolic risk^9,31^) due to increased adiposity. In this study, individuals with gradual weight gain had a higher proportion of poor health behaviors compared to those in the gradual-weight-loss group, but the association between weight gain and CVD risk showed a null association.

The results herein are supported by previous studies showing that weight loss was associated with increased health risk. A longitudinal study using data from the Helsinki Businessmen Study categorized individuals into four groups (constantly normal weight, constantly overweight, turning overweight, turning to a normal weight) according to their weight change across two time points (measured in 1974 and 2000) and the association with mortality risk in 2006 was assessed^11^. Participants who were overweight in midlife and turned to a normal weight in older age had a 1.9-fold (95% CI 1.2–3.0) higher risk of mortality compared to those with constantly normal weight. A study in Korea also found that sustained BMI loss increased the risk of all-cause mortality compared to those with a stable BMI^9^. In a US study with 36,051 participants, weight loss from middle to late adulthood was significantly associated with increased risk of heart disease mortality and all-cause mortality^7^. It was suggested that the intentionality of weight loss needs to be considered when evaluating the effect of weight loss on health risk. The ARIC study reported that middle-aged adults who unintentionally lost weight over the previous 3 years had a higher risk of coronary heart disease and ischemic stroke^10^. Another study showed that unintentional weight loss was associated with increased mortality rates^32^. It has been suggested that involuntary weight loss in middle-aged adults could be a warning sign for CVD^10^. Unintentional weight loss caused by underlying diseases may explain these paradoxical findings of the association between weight loss and increased CVD risk^33^. In this study, there was a higher proportion of individuals with diabetes and hypertension at baseline in the gradual-weight-loss group than in the other groups. Given that the unfavorable effect of weight loss on CVD risk was apparent in the lowest quartile of PA, muscle deficiency may have contributed, in part, to disease development^34^. Indeed, the observed changes in SMM over the 16-year follow-up period support this hypothesis. Meanwhile, in this study, the gradual-weight-loss group showed an inverse association with CVD risk in the third quartile of PA. Regular exercise has been shown to induce anti-inflammatory mechanisms, contributing to its beneficial effects on the prevention and treatment of CVD^35^. Muscle contraction during exercise causes skeletal muscle to secrete myokines, which mediate direct anti-inflammatory effects^36^. Since physical activity is involved in promoting myokine secretion^36^ and delaying muscle-mass loss^37^, maintaining regular physical activity can play an important role in the prevention of CVD.

Unlike previous studies, this study showed that the association between weight change and CVD risk depends on the level of PA. A study using ARIC study data reported that BMI, not weight change, affected the risk of atrial fibrillation and that the risk differed according to the level of PA^33^. The Aerobics Center Longitudinal Study suggested that improving fitness rather than weight change was more important in reducing the risk of CVD mortality^22^. When examining the relationship between obesity and CVD, it was stressed that PA should be taken into account^8^. Many studies consider PA as a covariate; however, few have reported the interaction effect of PA level. In addition, the lack of a standardized definition of PA makes direct comparisons difficult. In this study, PA level reflected physical labor as well as sports activity. Therefore, the null association at the highest PA level may indicate that sociodemographic characteristics according to PA level influenced the development of CVD. In our study, only baseline PA levels were considered. A systematic study of PA trajectories showed that consistently stable PA trajectory groups were more prevalent in adulthood^38^. In addition, many previous studies have assessed the relationship between physical activity and cardiovascular disease using baseline information alone^21^; however, some studies have reported that changes in physical fitness, in addition to baseline fitness, are associated with CVD mortality^22,39^. Thus, residual confounding effects from unmeasured physical activity at follow-up may affect the results, and some of the results may be overestimated. Also, the self-reporting of PA data may have introduced bias. Future studies should objectively measure both PA levels and changes thereof; the data may validate our results.

This study has several strengths and limitations. The results were obtained from a long-term observational study in a large population-based cohort. However, the study sample did not include the entire Korean population, which limits the generalizability of the results. While the possibility of measurement error exists, repeated measurements were used in an attempt to address this limitation in previous studies. Another limitation is that data on the intentionality of weight change were not collected. Therefore, further studies need to consider this to justify the association between weight loss and high CVD risk. In addition, only the baseline PA level was considered, so there may be residual confounding effects. Comorbidity such as dementia was not considered as a covariate. Due to the low incidence of specific CVDs, this analysis was limited and further investigation on a large scale is necessary. Although a given study^25^ evaluated the validities of self-reported CVD diagnoses by examining medical records, there were only 30 such cases. Accuracy issues associated with self-reporting of CVD can affect associations. In a previous study, the beneficial effect of weight gain in the underweight group and the negative effect of weight gain in the severely obese group were reported. However, in the current study, the subgroup could not be analyzed due to the very small size of the aforementioned subject groups. Finally, this study assessed the SMM changes associated with weight-change patterns and found that muscle loss was higher in the weight-loss group than in the other groups.

In summary, this study found that the association between weight loss and the incidence of CVD risk were dependent on the level of PA, and independent of baseline BMI. In addition, we observed a decrease in SMM with aging in all subjects, but the amount of decrease in SMM was higher in the weight loss group. Thus, these results imply that both healthy weight and appropriate PA are necessary to reduce the risk of incident CVD.

## Supplementary Information

Supplementary Information.

## Data Availability

The data described in the manuscript, code book, and analytic code will not be made available because the datasets used and/or analyzed during the current study are owned by a third party organization [The Korean Genome and Epidemiology Study-Ansan and Ansung study (KoGES); 4851-302]. These data are available online with permission from the Division of Epidemiology and Health Index of the Korea Centers for Disease Control and Prevention (KCDC).

## References

[1] Roth GA, Huffman MD, Moran AE (2015). Global and regional patterns in cardiovascular mortality from 1990 to 2013. Circulation.

[2] GBD 2017 Causes of Death Collaborators (2018). Global, regional, and national age-sex-specific mortality for 282 causes of death in 195 countries and territories, 1980–2017: A systematic analysis for the Global Burden of Disease Study 2017. Lancet.

[3] GBD 2017 DALYs and HALE Collaborators (2018). Global, regional, and national disability-adjusted life-years (DALYs) for 359 diseases and injuries and healthy life expectancy (HALE) for 195 countries and territories, 1990–2017: A systematic analysis for the Global Burden of Disease Study 2017. Lancet.

[4] World Health Organization, Cardiovascular diseases (CVDs) Fact sheet. https://www.who.int/news-room/fact-sheets/detail/cardiovascular-diseases-(cvds) (accessed 15 Jan 2020).

[5] Zheng W, McLerran DF, Rolland B (2011). Association between body-mass index and risk of death in more than 1 million Asians. N. Engl. J. Med..

[6] Liu X, Zhang D, Liu Y (2018). A J-shaped relation of BMI and stroke: Systematic review and dose-response meta-analysis of 4.43 million participants. Nutr. Metab. Cardiovasc. Dis..

[7] Chen C, Ye Y, Zhang Y, Pan XF, Pan A (2019). Weight change across adulthood in relation to all cause and cause specific mortality: Prospective cohort study. BMJ.

[8] Cho JH, Rhee EJ, Park SE (2019). Maintenance of body weight is an important determinant for the risk of ischemic stroke: A nationwide population-based cohort study. PLoS ONE.

[9] Cho IJ, Chang HJ, Sung JM, Yun YM, Kim HC, Chung N (2017). Associations of changes in body mass index with all-cause and cardiovascular mortality in healthy middle-aged adults. PLoS One.

[10] Stevens J, Erber E, Truesdale KP, Wang CH, Cai J (2013). Long- and short-term weight change and incident coronary heart disease and ischemic stroke: The Atherosclerosis Risk in Communities Study. Am. J. Epidemiol..

[11] Strandberg TE, Strandberg AY, Salomaa VV (2009). Explaining the obesity paradox: Cardiovascular risk, weight change, and mortality during long-term follow-up in men. Eur. Heart J..

[12] Zou H, Yin P, Liu L, Liu W, Zhang Z, Yang Y, Li W, Zong Q, Yu X (2019). Body-weight fluctuation was associated with increased risk for cardiovascular disease, all-cause and cardiovascular mortality: A systematic review and meta-analysis. Front. Endocrinol. (Lausanne)..

[13] Andruff H, Carraro N, Thompson A, Gaudreau P, Louvet B (2009). Latent class growth modelling: A tutorial. Tutor. Quant. Methods Psychol..

[14] Thompson AL, Koehler E, Herring AH (2016). Weight gain trajectories associated with elevated C-reactive protein levels in chinese adults. J. Am. Heart Assoc..

[15] Jeon J, Jung KJ, Jee SH (2019). Waist circumference trajectories and risk of type 2 diabetes mellitus in Korean population: The Korean genome and epidemiology study (KoGES). BMC Public Health.

[16] Janszky I, Romundstad P, Laugsand LE, Vatten LJ, Mukamal KJ, Mørkedal B (2016). Weight and weight change and risk of acute myocardial infarction and heart failure—the HUNT Study. J. Intern. Med..

[17] Flegal KM, Kit BK, Orpana H, Graubard BI (2013). Association of all-cause mortality with overweight and obesity using standard body mass index categories: A systematic review and meta-analysis. JAMA.

[18] Bagheri M, Speakman JR, Shabbidar S, Kazemi F, Djafarian K (2015). A dose-response meta-analysis of the impact of body mass index on stroke and all-cause mortality in stroke patients: A paradox within a paradox. Obes. Rev..

[19] Roh E, Choi KM (2020). Health consequences of sarcopenic obesity: A narrative review. Front. Endocrinol. (Lausanne)..

[20] Jakicic JM (2009). The effect of physical activity on body weight. Obesity (Silver Spring).

[21] Li J, Siegrist J (2012). Physical activity and risk of cardiovascular disease—A meta-analysis of prospective cohort studies. Int. J. Environ. Res. Public Health..

[22] Lee DC, Sui X, Artero EG (2011). Long-term effects of changes in cardiorespiratory fitness and body mass index on all-cause and cardiovascular disease mortality in men: The Aerobics Center Longitudinal Study. Circulation.

[23] Kim Y, Han BG, KoGES Group (2017). Cohort profile: The Korean Genome and Epidemiology Study (KoGES) Consortium. Int. J. Epidemiol..

[24] Kim NH, Seo JA, Cho H, Seo JH, Yu JH, Yoo HJ, Kim SG, Choi KM, Baik SH, Choi DS, Shin C, Cho NH (2016). Risk of the development of diabetes and cardiovascular disease in metabolically healthy obese people: The Korean Genome and Epidemiology Study. Medicine (Baltimore).

[25] Baik I, Cho NH, Kim SH, Shin C (2013). Dietary information improves cardiovascular disease risk prediction models. Eur. J. Clin. Nutr..

[26] Jones BL, Nagin DS, Roeder K (2001). A SAS procedure based on mixture models for estimating developmental trajectories. SMR..

[27] Costanzo S, Di Castelnuovo A, Donati MB, Iacoviello L, de Gaetano G (2010). Alcohol consumption and mortality in patients with cardiovascular disease: A meta-analysis. J. Am. Coll. Cardiol..

[28] Lee J, Lee C, Min J (2019). Development of the Korean Global Physical Activity Questionnaire: Reliability and validity study. Glob. Health Promot..

[29] Knowles R, Carter J, Jebb SA, Bennett D, Lewington S, Piernas C (2021). Associations of skeletal muscle mass and fat mass with incident cardiovascular disease and all-cause mortality: A prospective cohort study of UK Biobank participants. J. Am. Heart Assoc..

[30] Jones NR, Taylor KS, Taylor CJ, Aveyard P (2019). Weight change and the risk of incident atrial fibrillation: A systematic review and meta-analysis. Heart.

[31] Gordon-Larsen P, Koehler E, Howard AG (2014). Eighteen year weight trajectories and metabolic markers of diabetes in modernising China. Diabetologia.

[32] Gregg EW, Gerzoff RB, Thompson TJ, Williamson DF (2003). Intentional weight loss and death in overweight and obese U.S. adults 35 years of age and older. Ann. Intern. Med..

[33] Huxley RR, Misialek JR, Agarwal SK (2014). Physical activity, obesity, weight change, and risk of atrial fibrillation: The Atherosclerosis Risk in Communities study. Circ. Arrhythm Electrophysiol..

[34] Carbone S, Canada JM, Billingsley HE, Siddiqui MS, Elagizi A, Lavie CJ (2019). Obesity paradox in cardiovascular disease: Where do we stand?. Vasc. Health Risk Manag..

[35] Tao L, Bei Y, Zhang H, Xiao J, Li X (2015). Exercise for the heart: Signaling pathways. Oncotarget.

[36] Leal LG, Lopes MA, Batista ML (2018). Physical exercise-induced myokines and muscle-adipose tissue crosstalk: A review of current knowledge and the implications for health and metabolic diseases. Front. Physiol..

[37] Arthur ST, Cooley ID (2012). The effect of physiological stimuli on sarcopenia; impact of Notch and Wnt signaling on impaired aged skeletal muscle repair. Int. J. Biol. Sci..

[38] Lounassalo I, Salin K, Kankaanpää A, Hirvensalo M, Palomäki S, Tolvanen A, Yang X, Tammelin TH (2019). Distinct trajectories of physical activity and related factors during the life course in the general population: A systematic review. BMC Public Health.

[39] Mok A, Khaw KT, Luben R, Wareham N, Brage S (2019). Physical activity trajectories and mortality: Population based cohort study. BMJ.

