# Supplementary material for: Association between long-term weight-change trajectory and cardiovascular disease risk by physical activity level
Source: Sci Rep. 2022 Aug 12;12:13754. doi: 10.1038/s41598-022-17765-0 (PMC9374698; doi:10.1038/s41598-022-17765-0)
Supplement: Supplementary file 1 — Supplementary Information. [file 41598_2022_17765_MOESM1_ESM.pdf]

Association between Long-term Weight-change Trajectory and Cardiovascular Disease Risk by Physical Activity Level

Hye Ah Lee[1], Hyesook Park[2]

eFigure 1. Association between weight trajectory and incident cardiovascular disease by sex-specific quartile of physical activity-level and body-mass-index status at baseline.

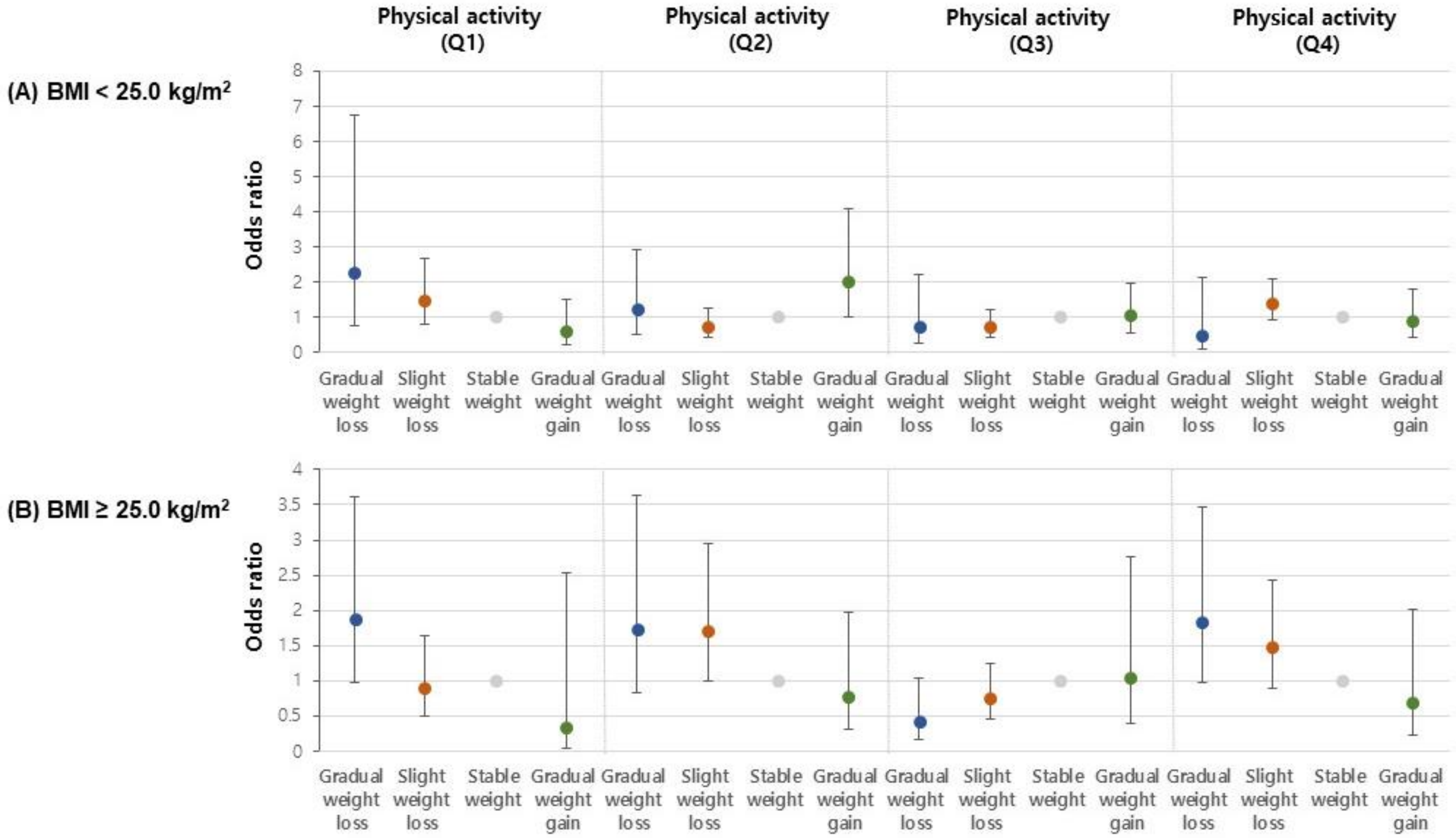

BMI, body mass index; Q, quartile.
Adjusted hazard ratios with 95% confidence interval were calculated with adjustment for sex, age, rural residence, educational level, body mass index, alcohol intake, current smoking, history of diabetes, history of hypertension, history of dyslipidemia, history of arthritis, and skeletal muscle mass change.

eTable 1. Comparison of the model fit results for candidate models via group-based trajectory modeling

Number of groups	Polynomial order	BIC
1	3	-145020.9
2	3 3	-135246.6
3	3 3 3	-130572.4
4	3 3 3 3	-128078.7
4	3 3 3 2	-128079.9
4	3 3 2 2	-128078.0
4	3 2 2 2	-128076.4
4	2 2 2 2	-128072.1
4	2 2 2 1	-128083.3
4	2 2 1 2	-128068.8
4	2 2 1 3	-130603.3
4	2 1 1 2	-128118.6

BIC, Bayesian information criterion.

eTable 2. Adjusted hazard ratio of incident CVD and all-cause mortality associated with weight trajectory by physical activity level, in men

Outcome	Weight-change Groups	Sex-specific quartiles of PA											
		PA Q1 (low)			PA Q2			PA Q3			PA Q4 (high)		
		N	HR	95% CI	N	HR	95% CI	N	HR	95% CI	N	HR	95% CI
All CVD	gradual weight loss	54	1.85	0.76-4.48	86	1.17	0.55-2.51	73	0.38	0.13-1.09	60	0.87	0.30-2.48
	slight weight loss	311	1.08	0.57-2.04	437	1.09	0.66-1.80	374	0.67	0.41-1.09	399	1.50	0.96-2.33
	stable weight	301	1.00		439	1.00		410	1.00		358	1.00	
	gradual weight gain	80	1.01	0.36-2.78	103	1.31	0.63-2.72	122	0.79	0.39-1.59	118	0.79	0.38-1.61
Fatal CVD	gradual weight loss	54	1.12	0.07-18.97	86	NA	NA	73	NA	NA	60	NA	NA
	slight weight loss	311	0.86	0.11-6.93	437	NA	NA	374	0.97	0.25-3.80	399	1.82	0.70-4.77
	stable weight	301	1.00		439	1.00		410	1.00		358	1.00	
	gradual weight gain	80	NA	NA	103	NA	NA	122	0.75	0.08-6.89	118	1.17	0.23-5.88
Non-fatal CVD	gradual weight loss	54	2.15	0.87-5.30	86	1.1	0.51-2.36	73	0.42	0.15-1.22	60	1.01	0.35-2.92
	slight weight loss	311	1.05	0.53-2.08	437	1.00	0.60-1.66	374	0.65	0.39-1.09	399	1.47	0.90-2.38
	stable weight	301	1.00		439	1.00		410	1.00		358	1.00	
	gradual weight gain	80	1.12	0.40-3.13	103	1.31	0.63-2.72	122	0.85	0.42-1.72	118	0.82	0.38-1.76
All-cause death	gradual weight loss	54	2.53	0.91-6.99	86	1.31	0.45-3.78	73	1.14	0.47-2.76	60	1.46	0.70-3.05
	slight weight loss	311	1.66	0.81-3.41	437	1.26	0.67-2.39	374	1.03	0.56-1.89	399	1.39	0.90-2.14
	stable weight	301	1.00		439	1.00		410	1.00		358	1.00	
	gradual weight gain	80	0.46	0.10-2.14	103	1.18	0.38-3.71	122	0.71	0.24-2.09	118	1.50	0.81-2.79

CVD, cardiovascular disease; PA, physical activity; HR, hazard ratio; 95% CI, 95% confidence interval.
Hazard ratios with 95% confidence intervals were calculated with adjustment for sex, age, rural residence, educational level, body mass index, alcohol intake, current smoking, history of diabetes, history of hypertension, history of dyslipidemia, history of arthritis, and skeletal muscle mass change.

eTable 3. Adjusted hazard ratio of incident CVD and all-cause mortality associated with weight trajectory by physical activity level, in women

Outcome	Weight-change Groups	Sex-specific quartiles of PA											
		PA Q1 (low)			PA Q2			PA Q3			PA Q4 (high)		
		N	HR	95% CI	N	HR	95% CI	N	HR	95% CI	N	HR	95% CI
All CVD	gradual weight loss	89	**2.95**	**1.51-5.76**	97	1.22	0.52-2.87	93	1.02	0.40-2.60	91	1.72	0.90-3.27
	slight weight loss	336	1.03	0.58-1.83	413	1.14	0.64-2.02	448	0.82	0.47-1.42	459	1.38	0.88-2.15
	stable weight	370	1.00		473	1.00		499	1.00		421	1.00	
	gradual weight gain	64	0.38	0.09-1.64	93	1.02	0.41-2.52	96	1.63	0.73-3.66	64	0.98	0.38-2.53
Fatal CVD	gradual weight loss	89	2.05	0.50-8.44	97	0.69	0.06-7.95	93	0.72	0.05-11.21	91	1.31	0.27-6.36
	slight weight loss	336	0.74	0.20-2.77	413	0.36	0.05-2.42	448	1.00	0.15-6.83	459	1.11	0.33-3.75
	stable weight	370	1.00		473	1.00		499	1.00		421	1.00	
	gradual weight gain	64	1.29	0.13-12.66	93	NA	NA	96	NA	NA	64	1.30	0.13-13.00
Non-fatal CVD	gradual weight loss	89	**3.30**	**1.55-6.99**	97	1.17	0.47-2.89	93	1.01	0.37-2.75	91	1.73	0.85-3.51
	slight weight loss	336	1.05	0.55-1.97	413	1.15	0.63-2.09	448	0.85	0.48-1.52	459	1.32	0.82-2.12
	stable weight	370	1.00		473	1.00		499	1.00		421	1.00	
	gradual weight gain	64	0.48	0.11-2.06	93	1.09	0.44-2.73	96	1.73	0.77-3.92	64	0.83	0.29-2.37
All-cause death	gradual weight loss	89	1.56	0.62-3.90	97	1.11	0.42-2.90	93	2.12	0.63-7.16	91	1.75	0.88-3.48
	slight weight loss	336	0.84	0.39-1.81	413	0.61	0.28-1.32	448	1.34	0.58-3.10	459	0.68	0.39-1.19
	stable weight	370	1.00		473	1.00		499	1.00		421	1.00	
	gradual weight gain	64	1.80	0.57-5.69	93	0.77	0.17-3.43	96	2.03	0.54-7.66	64	1.46	0.55-3.90

CVD, cardiovascular disease; PA, physical activity; HR, hazard ratio; 95% CI, 95% confidence interval.
Hazard ratios with 95% confidence intervals were calculated with adjustment for sex, age, rural residence, educational level, body mass index, alcohol intake, current smoking, history of diabetes, history of hypertension, history of dyslipidemia, history of arthritis, and skeletal muscle mass change.

eTable 4. Association between weight trajectory and incident cardiovascular disease by sex-specific quartile of physical activity-level in low muscle and high fat subjects.

Outcome	Weight-change Groups	Sex-specific quartiles of PA											
		PA Q1 (low)			PA Q2			PA Q3			PA Q4 (high)		
		N	HR	95% CI	N	HR	95% CI	N	HR	95% CI	N	HR	95% CI
All CVD	gradual weight loss	24	**5.05**	**1.23-20.67**	32	1.72	0.65-4.54	31	0.89	0.22-3.70	23	0.20	0.02-2.40
	slight weight loss	128	2.25	0.73-6.99	159	0.74	0.36-1.50	130	1.34	0.54-3.34	108	**3.76**	**1.17-12.12**
	stable weight	133	1.00		157	1.00		128	1.00		84	1.00	
	gradual weight gain	20	1.57	0.16-15.27	26	0.56	0.15-2.13	29	1.34	0.35-5.10	12	**6.58**	**1.08-40.08**
All-cause death	gradual weight loss	24	0.88	0.20-3.84	32	0.31	0.03-3.09	31	1.62	0.23-11.24	23	0.24	0.05-1.20
	slight weight loss	128	0.76	0.27-2.12	159	0.70	0.25-2.00	130	0.59	0.11-3.22	108	0.56	0.24-1.33
	stable weight	133	1.00		157	1.00		128	1.00		84	1.00	
	gradual weight gain	20	0.73	0.09-6.06	26	NA	NA	29	2.40	0.32-18.30	12	0.65	0.08-5.25

CVD, cardiovascular disease; PA, physical activity; HR, hazard ratio; 95% CI, 95% confidence interval.
Hazard ratios with 95% confidence intervals were calculated with adjustment for sex, age, rural residence, educational level, body mass index, alcohol intake, current smoking, history of diabetes, history of hypertension, history of dyslipidemia, history of arthritis, and skeletal muscle mass change.
